# Supplementary material for: Factors Associated with Older People’s Anxiety Symptom Positioning after COVID-19: Cross-Sectional Findings from a Canadian Sample
Source: Healthcare (Basel). 2024 Sep 13;12(18):1837. doi: 10.3390/healthcare12181837 (PMC11431020; doi:10.3390/healthcare12181837)
Supplement: Supplementary file 1 [file healthcare-12-01837-s001.zip › healthcare-3182074-supplementary.pdf]

STUDY INFORMATION AND INFORMED CONSENT LETTER

Title of the study: Mentally Healthy Living After Social Distancing – A study of older Canadians
Ethics Approval: Pro00118512

Principal Investigator: Dr. Gail Low
Associate Professor
Faculty of Nursing
University of Alberta
Edmonton, AB

Invitation to Participate: You are invited to participate in this RTOERO Foundation funded Canada-wide mental health research study because 1) you are 60+ years of age, and you are 2) a Qualtrics survey research Consumer Panel Member. Qualtrics is reaching out to its panel members to help us learn about mentally healthy living during this time of social distancing. Your thoughts and opinions are especially valuable and will be key to the success of the research project.

Purpose of the Study: Throughout the COVID-19 pandemic, Canadians 60+ years of age have been the most likely to get infected with this life-threatening virus. This virus impacts the physical, social, and mental health of older Canadians. Since March 2020, when the pandemic was declared in Canada, social distancing has been necessary. For many older people, this has meant living alone at home in isolation, with moderate to severe anxiety, fear, and loneliness. Many virtual (online) expert-driven mental health supports have been developed to help people cope with pandemic-related stresses, but most older Canadians do not see them as helpful nor as meeting their particular needs. Older people have been reluctant to seek in-person support services for fear of exposure to the COVID-19 virus. They also typically prefer to manage personal mental health concerns on their own. Mental health is also still a taboo subject.

From this research, we want to learn what has worked well for older Canadians who are thriving or doing well or perhaps just getting by despite this pandemic. What we'd like to learn from you is (1) mentally healthy living strategies that you are using to ease their way back into society, and (2) how these strategies reduce any age-related anxiety, stress, and other mental effects of the pandemic, and (3) what advice you would give other older Canadians who may be feeling lonely and isolated.

Participation: If you wish to participate in this study, please complete the attached survey. The survey should take you approximately 15–20 minutes to complete. You do not have to answer any questions that you do not want to answer. Once you have completed the survey, choose the "submit" button.

We would appreciate receiving it by the end of [name of 4th month after the survey is launched]. Qualtrics will send notices of reminder to Consumer Panel Members who have responded 'YES' to taking part by clicking on the survey hyperlink and have not yet submitted a survey. Three notices of reminder will be sent 2 weeks and 12 weeks after the survey is launched, and week 16 (one or two days before closing).

Benefits: Culinary and digital artists will put summaries of our findings into a cookbook-style recipe book. This book will feature anxiety-lessening strategies, sage advice, and simple healthy comfort food recipes. Mental health matters should be palatable matters. A recipe book with local ingredients or insights and advice from older Canadians is community-based action that may help isolated older Canadians feel connected and part of a larger community of recovery. Good mental health information tailored for and understandable to older Canadians could help to reduce social isolation and anxiety. We will ask cookbook users about these benefits in a separate over-time study.

Risks: This is a project about mental health. Mental health is still a taboo subject. You may feel emotional upset when you are completing our anxiety questionnaire, even though it is a short one. If you do, please reach out to a family member or friend or to your family doctor to seek further support. The people in our lives who matter most can help us feel less alone and give us strength.

We also have a list of Canada-wide toll-free mental health support lines (https://ccsmh.ca/wp-content/uploads/2020/04/Mental-Health-Support-Lines-for-Seniors-in-Canada.pdf), compiled by the Canada Coalition for Seniors' Mental Health (local crisis line information: 1-833-456-4566).

Confidentiality and Anonymity: The information that you will share will remain strictly confidential and will be used solely for the purposes of this research. The only people who will have access to the research data are the four research team professors from the University of Alberta, Simon Fraser University and Memorial University and the research assistant helping them to analyze surveys and to generate findings and summaries of findings for recipe book development. Your answers to open-ended questions may be used verbatim in presentations and publications, but neither you (nor your organization) will be identified.

In order to minimize the risk of security breaches and to help ensure your confidentiality, we recommend that you use standard safety measures such as signing out of your account, closing your browser and locking your screen or device when you are no longer using them / when you have completed the study. Because Qualtrics, our survey data collector, is located in the continental USA, the data is also subject to USA privacy legislation. Results will be published in pooled (aggregate) format. We will be asking you about your age, sex at birth, gender, education, marital status, and general health, and province. This will help us to see whether mentally healthy living strategies and their impacts on anxiety differ among, for example, men and women, and how healthy you feel. Anonymity is guaranteed. Your name, email or IP addresses will not be available to the research team.

Data Storage: Electronic copies of the survey dataset will be encrypted and stored on a password protected computer in the department of Nursing at the University of Alberta for 5 years.

Compensation (or Reimbursement): Qualtrics compiles a list of Consumer Panel Members who have responded 'YES' to taking part in our survey by clicking on the survey hyperlink. All Responders are assigned a unique identifier number for compensation and full survey access.

Responders would automatically be taken to a study information and informed consent 'Landing Page'. Your number is kept separate from your survey responses. You will be compensated the amount you agreed upon before you entered into the survey. If you choose to withdraw from the survey at any time, you will still receive this compensation.

Voluntary Participation: You are under no obligation to participate and if you choose to participate, you may refuse to answer questions that you do not want to answer. Qualtrics records all of your survey responses. Should you choose to withdraw partway through the electronic survey, simply close your survey link. Once you 'hit submit', we assume that you have submitted the survey data that you wanted to submit. Incomplete surveys about anxiety and mental health strategies will not be used for analysis. Where possible, we will compare characteristics to see whether submitters differ from non-submitters by age, sex at birth, gender, education, marital status, and general health and province.

Information about the Study Results:
At the end of this study, you will be able to access an electronic or hard copy of the cookbook through the RTOERO Foundation [info@rtoero.ca or toll-free at 1-800-361-9888].

Contact Information: If you have any questions or require more information about the study itself, you may contact the researcher by email (gail.low@ualberta.ca).

The plan for this study has been reviewed by a Research Ethics Board at the University of Alberta. If you have any questions regarding your rights as a research participant or how the research is being conducted, you may contact the Research Ethics Office at reoffice@ualberta.ca.

Please keep or print a copy of this form for your records.

Completion and submission of the survey means your consent to participate.
